# Structure, Mössbauer, electrical, and γ-ray attenuation-properties of magnesium zinc ferrite synthesized co-precipitation method

**DOI:** 10.1038/s41598-022-17311-y

**Published:** 2022-09-15

**Authors:** Hesham M. H. Zakaly, Shams A. M. Issa, H. A. Saudi, Gharam A. Alharshan, M. A. M. Uosif, A. M. A. Henaish

**Affiliations:** 1 Institute of Physics and Technology, Ural Federal University, 620003 Ekaterinburg, Russia; 2 Physics Department, Faculty of Science, Al-Azhar University, Assiut Branch, Assiut, 71524 Egypt; 3 Physics Department, Faculty of Science, University of Tabuk, Tabuk, 71451 Saudi Arabia; 4 Department of Physics, Faculty of Science, Al-Azhar University (Girls’ Branch), Nasr City, Egypt; 5 Physics Department, College of Science, Princess Nourah Bint, Abdulrahman University, P. O. Box. 84428, Riyadh, 11671 Saudi Arabia; 6 Physics Department, College of Science, Jouf University, P. O. 2014, Sakaka, Al-Jouf Saudi Arabia; 7 Physics Department, Faculty of Science, Tanta University, Tanta, 31527 Egypt

**Keywords:** Materials science, Nuclear physics

## Abstract

For technical and radioprotection causes, it has become essential to find new trends of smart materials which used as protection from ionizing radiation. To overcome the undesirable properties in lead aprons and provide the proper or better shielding properties against ionizing radiation, the tendency is now going to use ferrite as a shielding material. The co-precipitation method was utilized to prevent any foreign phases in the investigated MZN nano-ferrite. X-ray diffraction (XRD) and Fourier transmission infrared spectroscopy (FTIR) methods were used to analyze the manufactured sample. As proven by XRD and FTIR, the studied materials have their unique spinel phase with cubic structure Fd3m space group. The DC resistivity of Mg–Zn ferrite was carried out in the temperature range (77–295 K), and its dependence on temperature indicates that there are different charge transport mechanisms. The Mössbauer spectra analysis confirmed that the ferrimagnetic to superparamagnetic phase transition behaviour depends on Zn concentration. The incorporation of Zn to MZF enhanced the nano-ferrite density, whereas the addition of different Zn-oxides reduced the density for nano-ferrite samples. This variation in density changed the radiation shielding results. The sample containing high Zn (MZF-0.5) gives us better results in radiation shielding properties at low gamma, so this sample is superior in shielding results for charged particles at low energy. Finally, the possibility to use MZN nano-ferrite with various content in different ionizing radiation shielding fields can be concluded.

## Introduction

Although technological advancements have made life easier for humans, they have also had negative consequences, such as the rapid expansion of nuclear waste storage sites nuclear radiation use in our daily lives, which includes industries, medical diagnostic centers, nuclear reactors, food irradiation, nuclear research institutions, and medical diagnosis as well as therapy^1^. Because of their magnetic, electrical, optical, and mechanical properties, oxide-based samples such as nano-ferrites have received a great deal of attention in recent years^2–4^. As a result, these materials have the capacity to be used in a many applications, including medical diagnostics, rechargeable lithium batteries, high-frequency media, solar energy devices, magnetic fluids, and radiation shielding materials^5–7^. In this study, researchers used ferrites in the form of nanoparticles to discover that the physical and chemical characteristics of these ferrites in the nano-range are influenced by factors such as crystal size, energy band gap, surface, and bulk morphology^8,9^, amongst other things^10^.

One of the most famous magnetically soft spinel materials, Magnesium Zinc ferrite (MZF) in nano size, is an ecologically benign, non-toxic substance that absorbs visible light owing to its tiny bandgap, and that may be used as an attenuated material for gamma rays. A diverse collection of past studies demonstrates a continuing interest in radiation shielding against ionizing radiation^11–13^. Likewise, the pollution created by gamma-ray radiation is a severe concern in electronic, networking, and wireless equipment, demanding the study and development of radiation absorption materials^14–16^. The general idea of using spinel soft magnetic materials is depending on the magnetic and electric properties of this materials, such as the higher value of electrical conductivity, permittivity (σ_r_), and permeability (μ_r_) of the material^17^. Although, the use of a composite between polymer or a block of cement with magnetic ferrite as a filler is a smart way forward to enhance EMI shielding performance^18,19^.

It is necessary to limit the undesired emissions from materials/devices and external media in order to regulate and postpone the negative effects of ionizing radiation, as well as the detrimental influence on people. The capacity of shielding materials to deflect and absorb radiation is the primary function of these materials. We know that charge carriers in electrically conductive shielding materials cause reflection, and we can predict how this happens^20–22^. The absorption process is carried out by the usage of the magnetic and electric dipoles of shielding materials. Several shielding materials are used to conduct the absorption radiation; these materials were selected for their intelligent and promising radiation shielding properties, and they include carbon nanotubes^23^, graphene^24^, polymers^25^, BaTiO3^6,26^, PZTiO3^27^, and nano ferrite^28^.

This work involves synthesizing Mg_(1−x)_Zn_x_Fe_2_O_4_ (MZF) in Nanoscale via a chemical method and developed to use the magnetic ferrite to be used as filler materials which added to attenuate the gamma radiation, which has a fundamental contribution on absorption decay that lead to high-shielding effectiveness with high attenuation of ionizing radiation.

## Materials and methods

In the presence investigation of Mg_(1−x)_Zn_x_Fe_2_O_4_ samples where (x = 0.0, 0.10, 0.20, 0.30, 0.40 and 0.50) ferrite system were prepared using the Co-precipitation method^29,30^. The starting materials were MgCl_2_·6H_2_O, ZnCl_2_, and FeCL_3_·6H_2_O (1:2 molar-ratio) by addition 25% amonia-solution. The whole substance utilized was brought in from Oxford Lab and was of very high chemical purity (99.99%). Reagent. The ferrite system was prepared in a typical reaction,

$$\begin{aligned} & \left( {1 - {\text{x}}} \right){\text{MgCl}}_{2} \cdot 6{\text{H}}_{2} {\text{O}} + {\text{xZnCl}}_{2} + 2{\text{FeCl}}_{3} \cdot 6{\text{H}}_{2} {\text{O}} + 8{\text{NaOH}} \\ & \quad \to {\text{Mg}}_{{(1 - {\text{X}})}} {\text{Zn}}_{{({\text{X}})}} {\text{Fe}}_{2} {\text{O}}_{4} + 8{\text{NaCl}} + \left( {22 - 6{\text{X}}} \right){\text{H}}_{2} {\text{O}} \\ \end{aligned}$$

**Scheme 1:**
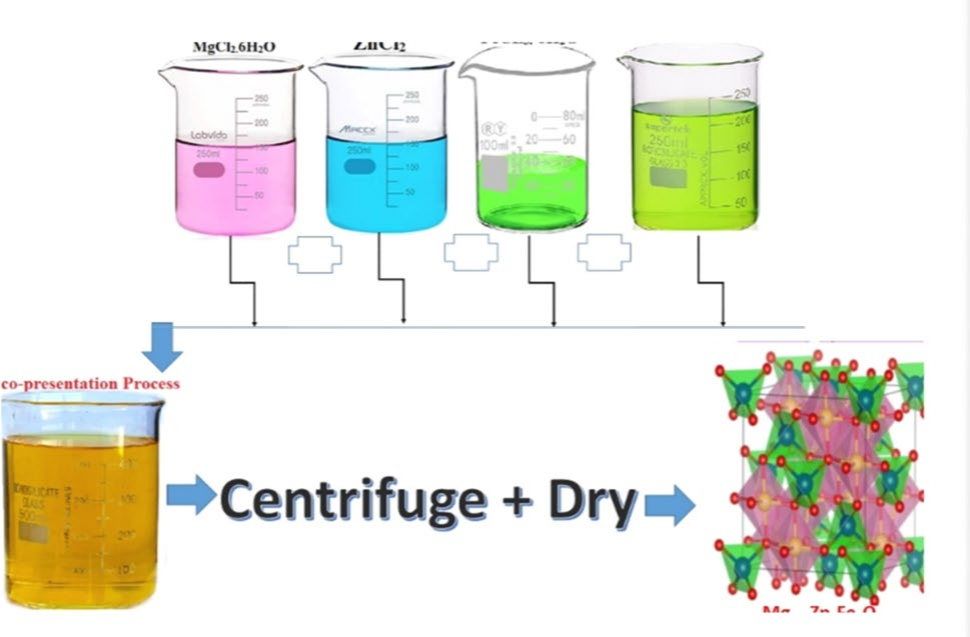
Schematic view of synthesis of Mg_(1−x)_Zn_x_Fe_2_O_4_ by Co-precipitation method.

The volume of the reaction mixture was combined under magnetic stirring during a continual gradual addition of 25 ml to a 25% ammonia solution, with the heating continuing for thirty minutes. A black precipitate was decanted and washed with 500 ml distilled water in a changing magnetic field (Scheme 1).

**Figure 1:**
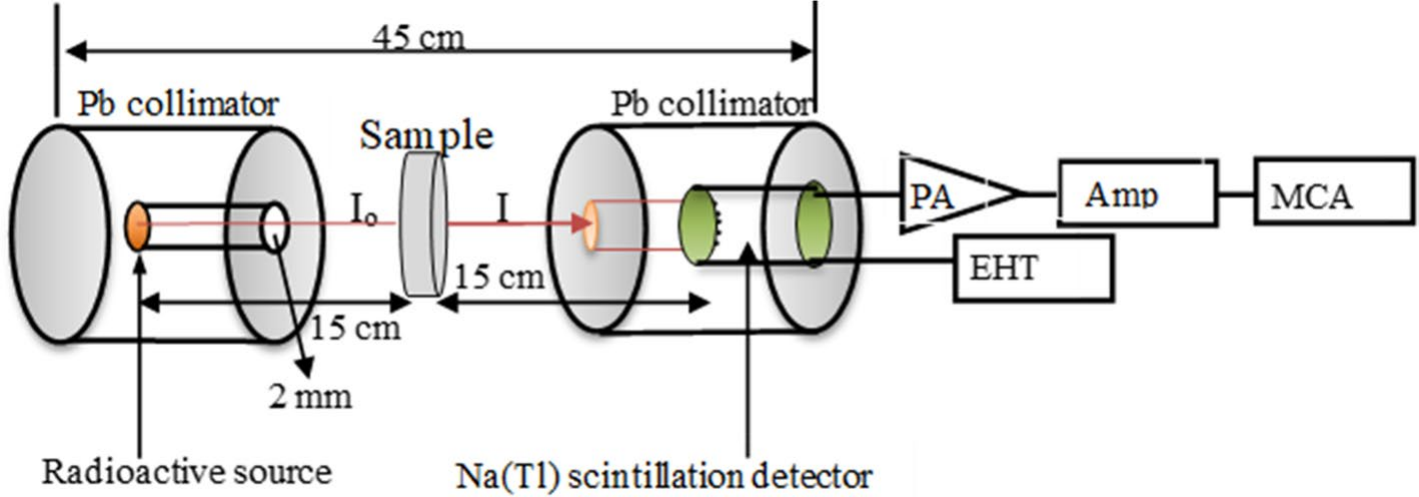
Radiation measurement setup.

The linear attenuation coefficients (µ) of ferrite samples have been measured experimentally using the narrow beam method in conjunction with a Pb-collimator. The collimated photons, which have varying energy, have interacted with several types of glass samples. Radiation measurements were performed with a NaI (Tl)-scintillation detector (Oxford model) with a 3–3-in. detection window, which was coupled to a multichannel analyzer^31^. The radioactive sources that were employed in the experiment were Ba-133 (81 and 356 keV, 1 µCi), Cs-137 (662 keV, 5 µCi), Co-60 (1173 and 1332 keV, 10 µCi), and Th-233 (911 and 2614 keV, 20 µCi). Figure 1 depicts the experimental setup, which includes the source, sample, and detector. The area beneath photopeak has been used to determine the photon intensity without and with absorber for each gamma-line in the experiment. The uncertainties were fewer than 1% of the total number of uncertainties. The spectra were analyzed utilize the Genie-2000 software, which was developed by Canberra.

## Results and discussions

### Structure properties

**Figure 2:**
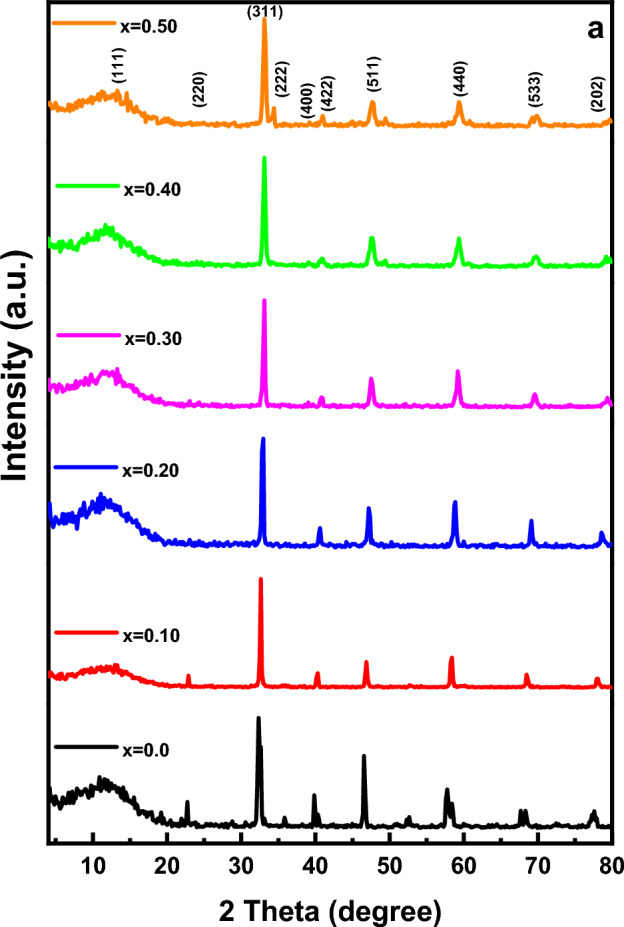
XRD-pattern of Mg_(1−x)_Zn_x_Fe_2_O_4_ samples where (x = 0.0, 0.10, 0.20, 0.30, 0.40 and 0.50).

X-ray diffraction patterns of the investigated samples annealed at 1100 °C were illustrated in Fig. 2. XRD patterns indicate single-phase cubic spinel structure with the main peak (311)^6,32–35^. It can confirm from the broad XRD peaks that the samples consist of ultrafine nano-sized particles. The crystallite size was estimated from XRD from the most intense peaks using Scherer's Eq. (1) and found in the range 35–45 nm. The calculated values of the lattice parameter (a), the crystallite size (t), interplanar distance (d), the X-ray density (d_x_) and the bulk density (d_B_), and the percentage of porosity (P) are also summarized in Table 1.

**Table 1:** Values of Lattice parameter a_exp_ (Å), crystallite size t (nm), interplanar distance d (nm), X-ray density d_x_ (g cm^−3^), Bulk density-d_B_ (g cm^−3^), Porosity P (%), Interchain separation R (nm), micro strain ɛ, dislocation density δ (nm^−2^), and distortion parameters-g for prepared Mg_(1−x)_Zn_x_Fe_2_O_4_ samples.

x	a_exp_	a_th_	t	d	d_x_	d_B_	P	R	ɛ	δ	g	Cation distributions
0	8.43	8.44	35.9	4.86	5.17	3.38	34.62	3.32	0.133	0.0014	1.868	(Mg_0.05_·Fe_0.95_) [Mg_0.95_·Fe_1.05_]
0.1	8.39	8.49	41.2	2.98	5.29	3.44	34.97	2.44	0.147	0.0018	1.480	(Mg_0.1_·Zn_0.1_ Fe_0.8_) [Mg_0.8_·Fe_1.2_]
0.2	8.36	8.52	44.8	2.54	5.03	3.31	34.19	2.21	0.182	0.0027	1.6722	(Mg_0.06_·Zn_0.2_·Fe_0.74_) [Mg_0.74_·Fe_1.26_]
0.3	8.30	8.5	39.3	2.43	4.92	3.25	33.94	2.35	0.238	0.0047	2.305	(Mg_0.07_·Zn_0.3_·Fe_0.64_) [Mg_0.63_·Fe_1.36_]
0.4	8.25	8.47	37.3	2.15	4.74	3.20	32.48	2.41	0.197	0.0032	1.894	(Mg_0.08_·Zn_0.4_·Fe_0.52_) [Mg_0.52_·Fe_1.48_]
0.5	8.22	8.37	36.0	1.72	4.54	3.11	31.49	2.49	0.195	0.0031	1.928	(Mg_0.05_·Zn_0.5_·Fe_0.45_) [Mg_0.45_·Fe_1.55_]

**Figure 3:**
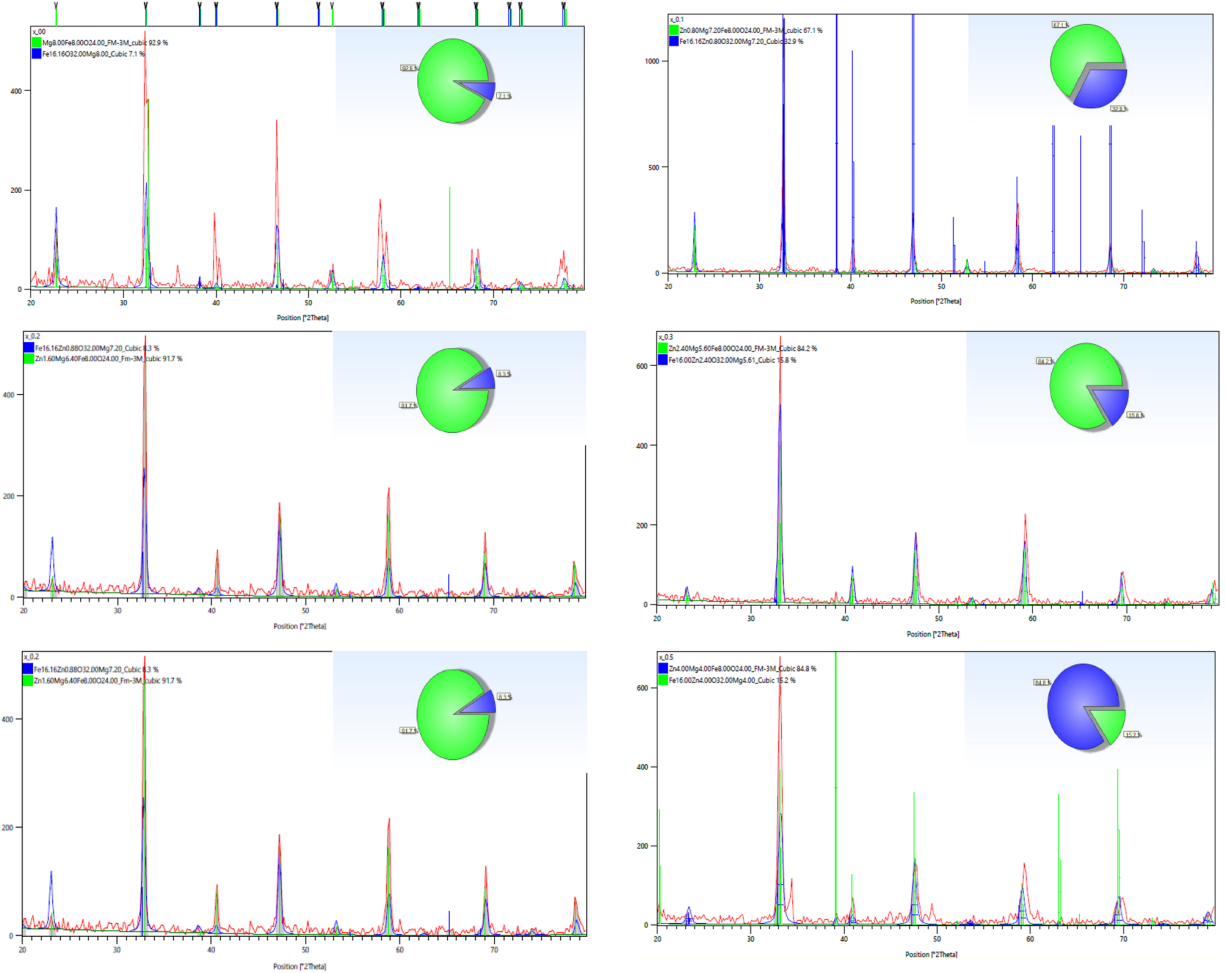
The matching high score plus for XRD patterns for all samples.

The matching high score plus of Mg_(1−x)_Zn_x_Fe_2_O_4_ samples are shown in Fig. 3. Diffraction peaks corresponding to diffraction planes (111), (220), (311), (222), (400), (422), (511), (440), (533) and (202) are indexed to single-phase with space group Fm-3m.

The structural and lattice parameter of Mg_(1−x)_Zn_x_Fe_2_O_4_ samples were determined based on the full width at half maximum-FWHM (β), Bragg angle (θ in radians), and Miller indices of each plane (h k l) of the diffraction peak. With the help of the following equations, we can determine the interplanar distance (d′), microstrain (ɛ), interchain separation (R), the crystallite size (d), dislocation density (δ), and distortion parameters (g)^36^:

1 $$t=\frac{k\lambda }{\beta\,\, \text{cos}({\theta }_{B})}$$

where k = 0.89; and λ wavelength of the X-ray for Cu–κα radiation = 1.541178 Å

2 $${a}_{exp}={d}{^{\prime}}\sqrt{{h}^{2}+{k}^{2}+{l}^{2}}$$

3 $${d}^{{\prime}}=\frac{\lambda }{2\text{sin}\,\,(\theta )}$$

4 $$R=\frac{5 \lambda }{8\,\,\text{sin}(\theta )}$$

5 $$\varepsilon =\frac{\beta\,\, \text{cos}(\theta )}{4}$$

6 $$g=\frac{\beta }{\text{tan}(\theta )}$$

7 $$d=\frac{k \lambda }{\beta\,\, \text{cos}(\theta )}$$

8 $$\delta =\frac{1}{{d}^{2}}$$

The structural parameters R, ɛ, d, δ, and g are calculated and tabulated in Table 1. The calculated data, shown in Table 1, shows that lattice parameter (a) values decrease with decreasing Mg content^37^. This decrease can be attributed to replacing Mg^2+^ ion with a smaller ionic radius (0.066 nm) with Zn^2+^ ion with a larger ionic radius (0.082 nm). Also, the unusual density behavior that grows up to x = 0.3 and then decreases may be attributed to the replacement of lighter Mg by heavier Zn atoms and the distribution of zinc concentration among sublattice and, therefore, the influence of condensation on the crystal structure^38^. The assessed values in Table 1 show that the Mg-Zn ferrite composition significantly reduces both XRD and bulk density. This is related to the replacement of Mg^2+^ ion with lower ionic radius (0.066 nm) by Zn^2+^ ion with a larger ionic radius (0.082 nm) Zn^2+^ ions in a spinel ferrite, on the other hand, have a significant affinity for tetrahedral interstitial spaces (A-sites) and may therefore replace both Mg^2+^ and Fe^3+^ ions in A-sites as given from The cation distribution. All of this demonstrates that the proportion of vacancies in the materials is increasing, which has an impact on packing density.

**Figure 4:**
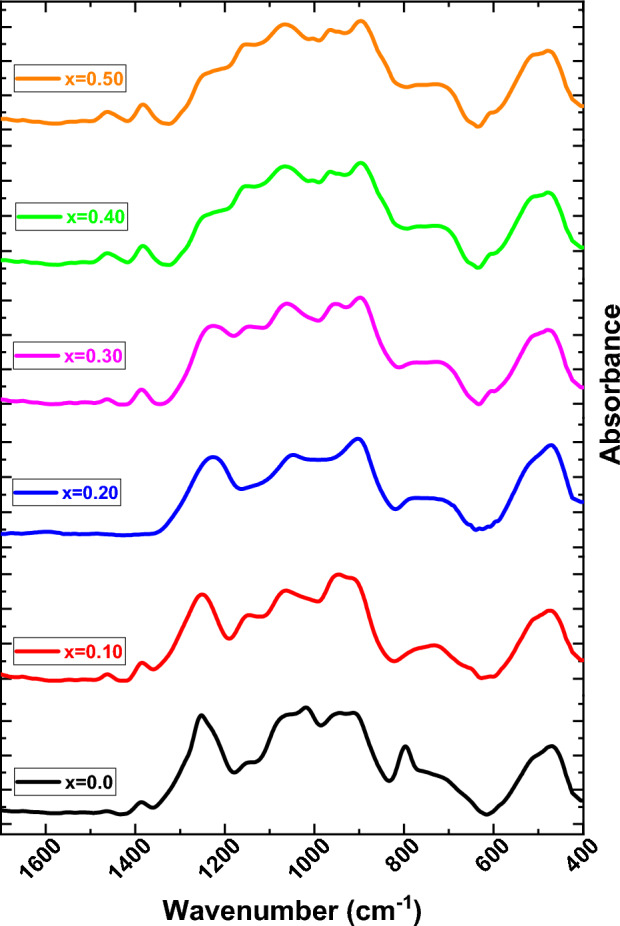
FTIR of Mg_(1−x)_Zn_x_Fe_2_O_4_ samples where (x = 0.0, 0.10, 0.20, 0.30, 0.40 and 0.50).

Figure 4 illustrates the FTIR spectra in the wavenumber range (400–1500 cm^−1^) at room temperature for Mg_(1−x)_Zn_x_Fe_2_O_4_ samples prepared using the co-precipitation method. It can notice that the higher frequency (υ_Tetra_) attributed to the intrinsic vibration of the tetrahedral complex around ≈ 600 cm^−1^, the lower frequency (υ_Octa_) attributed to the intrinsic vibration of the tetrahedral complex around ≈ 450 cm^−1^^39,40^. This change between two frequencies can be explained due to the change in Fe^3+^_O^−^ complex in the Mg_(1−x)_Zn_x_Fe_2_O_4_ ferrite system because of occupation possibility Zn^2+^ ions at of tetrahedral (A) sites, Fe ions partially occupy A-sites and B-sites. Mg^2+^ decreases at A sites and/or B-sites replacing Zn^2+^ ion, leading to the migration of some Fe-ions from B-sites to A-sites. It can be observed that the characteristic IR bands as shown distinguished bands near 1400 cm^−1^. Which is attributed to the stretching modes and H–O–H bending vibrations of the free or absorbed water^30^.

The force constants (FC) at the A and B-sites, which are dependent on the vibrational frequencies, are (F_Octa_), and (F_Tetra_), respectively, as given in Table 2. It can be seen that the force constant at the tetrahedral site is more extensive than that at the octahedral sites. The reduction in the force constant at the tetrahedral site after Zn^2+^ substitution in MgFe_2_O_4_ indicates that Zn^2+^ ions occupy the tetrahedral sites. $F=4\pi {c}^{2}{v}^{2}\mu$ was used to calculate the force constant of vibrating bonds, where c is the speed of light in space (cm/s), is the wavenumber of frequency, and is the decreased mass of Fe^3+^ and O^2−^ ions, which is given by $\mu =\frac{{m}_{o}*{m}_{Fe}}{{m}_{o}+{m}_{Fe}}$^39^.

**Table 2:** Values of Frequency (υ_Octa_, υ_Tetra_), (Velocity' Octa × 10^8^, Velocity' Tetra × 10^8^), (n) A and B-site, E (Octa) × 10^–4^, E (Tetra) × 10^8^, P (Octa) × 10^–4^, and P (Tetra) × 10^8^ for prepared Mg_(1−x)_-Zn_x_-Fe_2_O_4_ samples.

x	υ_Octa_	υ_Tetra_	Velocity' Octa	Velocity' Tetra	(n) B-site (Tetra)	(n) A-site	E (Octa)	E (Tetra)	P Octa	P Tetra
0	464.108	609.66	2.541	3.000	3.4723	5.1767	2.31	2.73	2.532	2.986
0.1	467.45	616.49	2.694	2.755	4.1752	6.1659	2.43	2.51	2.684	2.744
0.2	470.96	622.0055	2.602	2.969	7.1967	8.7087	2.37	2.70	2.593	2.957
0.3	472.62	623.15	2.633	3.000	3.59618	3.35327	2.39	2.73	2.623	2.987
0.4	482.9634	622.0055	2.572	3.030	3.5962	3.3533	2.34	2.76	2.563	3.019
0.5	484.2367	624.8857	2.663	3.092	1.477	2.0519	2.42	2.81	2.654	3.079

**Figure 5:**
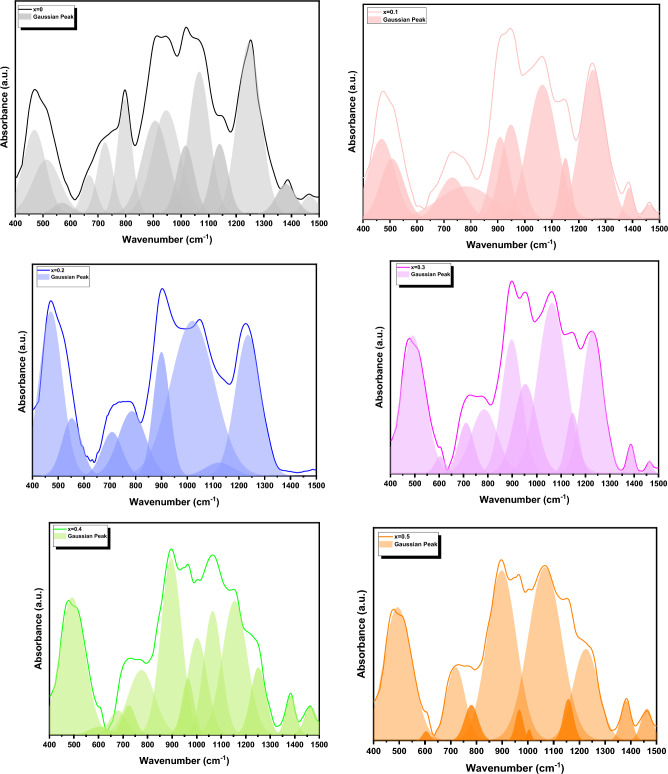
Gaussian deconvolution of FTIR spectrum of Mg_(1−x)_Zn_x_Fe_2_O_4_ samples where (x = 0.0, 0.10, 0.20, 0.30, 0.40 and 0.50).

It can show from Fig. 5 that there is an overlapping in the absorption band in FTIR spectra for all samples. Therefore, for more analysis and getting profound information about the changes in the structure and position of the absorption band which occur through the investigated samples by using means of the deconvoluted spectra via several Gaussians peaks ≈ (8–14 peaks). All the getting parameters which getting from FTIR deconvoluted peaks are illustrated in Table 3.

**Table 3:** The deconvolution parameter of the IR-spectra of the samples under investigation (C) represents the component band center, and (A) represents the relative area (percentage) of the component band.

	X = 0.0	X = 0.10	X = 0.20	X = 0.30	X = 0.40	X = 0.50
**G-1**						
C	468	467	470	–	492	493
A	6.07	4.21	6.64	–	9.62	10.02
**G-2**						
C	512	507	552	491	–	
A	4.75	2.84	1.95	8.17	–	
**G-3**						
C	569	610	–	603	604	604
A	0.61	0.016	–	0.44	0.47	0.18
**G-4**						
C	667	660	–	–	678	–
A	1.76	0.144	–	–	1.11	–
**G-5**						
C	724	730	708	710	723	718
A	3.53	2.19	1.56	1.9	1.06	3.68
**G-6**						
C	795	780	785	784	774	780
A	5.28	3.56	3.06	4.08	4.78	1.29
**G-7**						
C	906	908	899	897	896	898
A	7.88	3.08	3.54	6.58	10.1	13.23
**G-8**						
C	946	948	–	953	964	966
A	9.11	3.91	–	4.95	1.87	0.65
**G-9**						
C	1016	1000	1020	1062	1002	1004
A	3.76	3.02	11.8	11.62	5.26	0.138
**G-10**						
C	1065	1065	–	–	1065	1066
A	9.62	7.88	–	–	6.20	15.30
**G-11**						
C	1139	1150	1123	1147	1154	1157
A	3.73	1.33	0.65	1.86	9.15	1.12
**G-12**						
C	1246	1253	1234	1229	1251	1225
A	12.82	7.64	6.20	7.3	2.84	6.16
**G-13**						
C	1397	1387	–	1385	1384	1381
A	1.85	0.61	–	0.49	1.12	1.12
**G-14**						
C	1466	1462	–	1463	1462	1462
A	0.83	0.36	–	0.166	1.00	0.98

### Electrical properties

**Figure 6:**
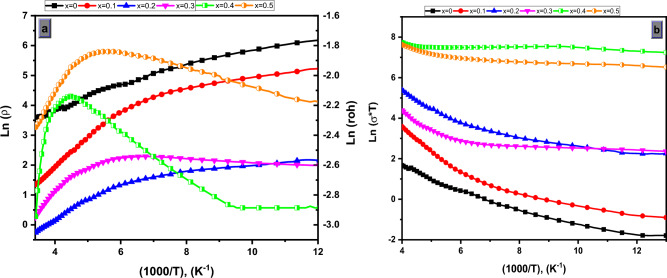
(Ln ρ), (Ln σ) Vs. (1000/T, (k^−1^)) of Mg_(1−x)_Zn_x_Fe_2_O_4_ samples where (x = 0.0, 0.10, 0.20, 0.30, 0.40 and 0.50).

Figure 6a,b show the dependence of electrical resistivity and conductivity for Mg_(1−x)_Zn_x_Fe_2_O_4_ ferrite system upon temperature ranges (77–295 K). It can clearly be noticed that the existence of two linear regions characterizes each conductivity curve which, can be attributed to the presence of different charge transport mechanisms^41,42^. The ln(σ) versus 1000/T plot shows a mono-linearity relationship to estimate the activation energy across the entire temperature range. Therefore, the activation energy (E_a_) was determined using the Arrhenius equation where the corresponding ln(σT) against 1000/T plot shows an approximately linear relationship as shown in Eq. (9)^43–45^.

9 $$\rho ={\rho }_{o }\,\,exp\frac{{E}_{a}}{ {K}_{B}T}$$

In this equation, ${\rho }_{o}$ is the resistivity at room temperature, $\Delta E$ is the activation energy in electron volts, k is the Boltzmann's constant, 8.625 × l0^–5^ eV/K, and T is the absolute temperature. There were two parallel conductivity processes with differing activation energies that were responsible for the change in slope in all curves. This shift in slope is often seen at temperatures that are close to the Curie temperature of the samples (Tc)^46–48^.

**Figure 7:**
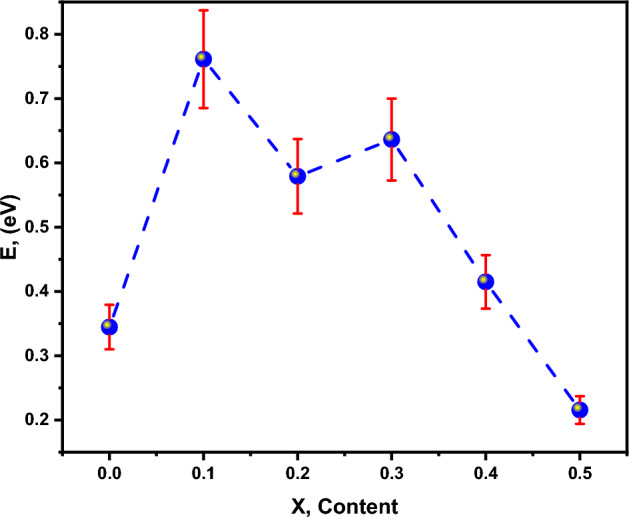
Activation energy of Mg–Zn ferrite system with different Mg-content-(x).

It was possible to compute the activation-energy of each sample within the observed temperature range at the slope of linear plots of resistivity. According to the results, the activation energy was determined to be ranged 0.21–0.76 eV, as shown in Table 4 and Fig. 7. It was discovered that increasing the Zn content in the system Mg_(1−x)_Zn_x_Fe_2_O_4_ ferrite up to x = 0.2 resulted in an increase in activation energy, and then decreases can be attributed to the theory of can be attributed to the presence of different charge transport mechanisms and the decrease this can be attributed to the theory of a change in activation energy is due to the splitting of the conduction band and the valence bands below (Tc) the higher value of activation energy at higher concentration of Zn indicate the strong blocking of the conduction mechanism between Fe^3+^ and Fe^2+^ ions^48^.

**Table 4:** ρ (at Rt); T_c_ (K), E_a1_ (eV) (from Ln ρ) and E_a2_ (eV) (from Ln σ*T) of investigated nano-ferrite samples.

X	ρ (at Rt)	T_c_ (K)	Activation energy E_a1_ (eV) (from Ln ρ)	Activation energy E_a2_ (eV) (from Ln σ*T)
0.0	36.75	153	0.34	0.51
0.10	3.88	136	0.76	0.93
0.20	0.77	195	0.57	0.68
0.30	1.29	170	0.63	0.98
0.40	0.052	225	0.41	0.57
0.50	0.096	184	0.21	0.43

### Mösbauer spectroscopy

**Figure 8:**
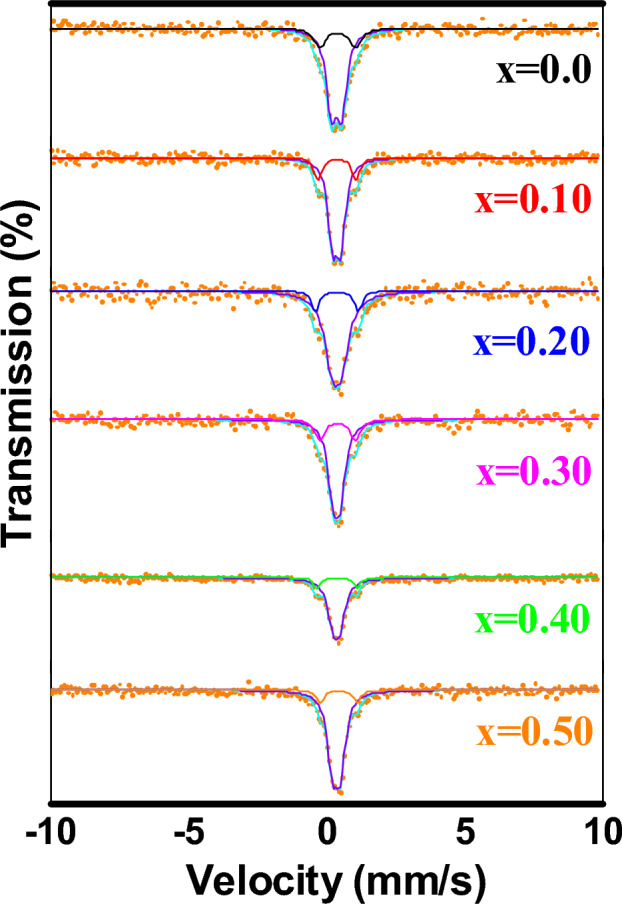
Fitted Mössbauer spectra for samples Mg(1−x) Zn(x)Fe_2_O_4_.

For all samples weighing 0.015 g, homogenous and well milled Mössbauer spectroscopy measurements were performed. The sequential decay of the ^57^Co source produced 14.4 keV rays (5 mCi). All measurements were performed over a speed range of ± 10 mm s at room temperature (RT), and spectral data were fitted using Lorentzian line shapes. The Mösbauer spectra of Mg_1−x_Zn_x_Fe_2_O_4_ were acquired at (RT) and fitted using were fitted using Lorentzian line shapes (Fig. 8). Illustrated the hyperfine parameters, isomer shift (I.S.), magnetic hyperfine field (Hhf), quadrupole shift (Q.S.), relative area (A0), and line width (Г). Analyzing the Mösbauer spectra for all recorded spectra (x = 0–0.5) is characteristic by splitting doublets, which attributed to the presence of Fe^3+^ ion at the tetrahedral and octahedral site and confirmed the superparamagnetic behavior of the Mg–Zn ferrite samples^49,50^.

A single sextet (B) in addition to superparamagnetic doublet were observed; this indicates relaxation effects, i.e., the presence of ions only in the octahedral B site whereas the magnetic sextet of A site vanishes. However, the possibility of occupying Fe^3+^ ions in both A and B sites can slightly affect the magnetic hyperfine field values, quadrupole shift isomer shift, and connection to the substitution of Zn in the Mg-ferrite composition. For all samples, the centers of the Zeeman lines are not changed (0.446 for sextet (B) and 0.431 for doublet), denoting that replacement of Mg^2+^ by Zn^2+^ ions did not change the site symmetry.

The fitted parameters given in (Table 5) show the fitted Mossbauer parameters isomer shift (δ), quadrupole splitting (ΔE_Q_), and Area (A) The isomer shift of sextet (B) is assigned to the iron ions at the B site, due to difference in Fe^3+^–O^2−^ internuclear separation^51–53^. Area of under Mössbauer spectra for sextet (B) systematically decrease as the Zn-content increases in B site. Attributed to the increase in the weak paramagnetic character (Zn ions) while the ferromagnetic character is decreasing (Fe ion), i.e., weakens the inter sublattice (AB) interactions between Fe ions. As the particle sizes are small, the crystallization will be imperfect. The ΔE_Q_ values decrease with increasing Zn content indicating less local distortion at the B sites of ferrite structure^54^. The growth of superparamagnetic doublet due to decreased particle size with increasing Zn content which means a reduction in the bulk magnetization. Due to a large number of nonmagnetic nearest neighbors, the central doublet can be attributed to the magnetically isolated ions which do not contribute to the long-range magnetic ordering^55,56^.

**Table 5:** Mossbauer parameters for Mg(1−x) Zn(x)Fe_2_O_4_. (All values in the table are in units of mm S^−1^ and Relative errors − 0.01 mm S^−1^).

X	Component	Isomer shift δ (mm s^−1^)	ΔEQ (mm s^−1^)	Area (%)
0.0	Sextet (B)	0.349	0.371	79.1
	Double	0.392	1.358	17.9
0.10	Sextet (B)	0.349	0.354	78.9
	Double	0.362	1.346	18.9
0.20	Sextet (B)	0.349	0.351	83.9
	Double	0.358	1.281	13.8
0.30	Sextet (B)	0.349	0.349	75.8
	Double	0.377	1.281	22.7
0.40	Sextet (B)	0.349	0.341	84.9
	Double	0.367	1.339	13.8
0.50	Sextet (B)	0.352	0.341	87.6
	Double	0.424	1.339	11.2

### Radiation shielding properties

Transmissions (T) have been calculated using the following formula based on photon intensities (I) and glass thickness (t) for a variety of ferrite samples at various energies^57,58^:

10 $$I={I}_{0 }\,\,{e}^{-\mu t}$$

**Figure 9:**
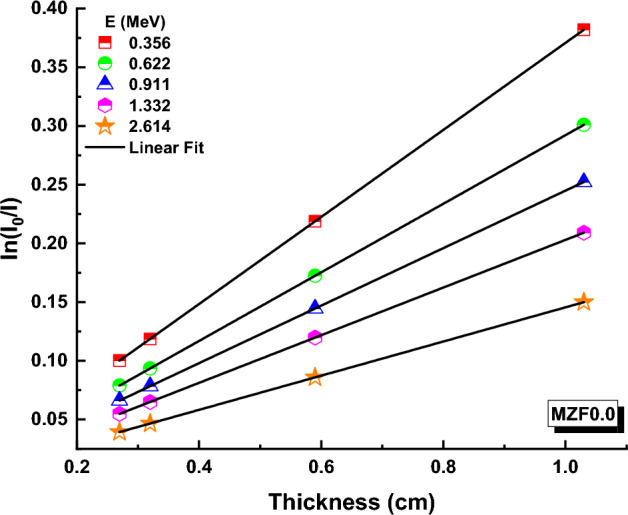
Transmission variation against thickness (x) values for MZF0.0 nano-ferrite sample.

**Figure 10:**
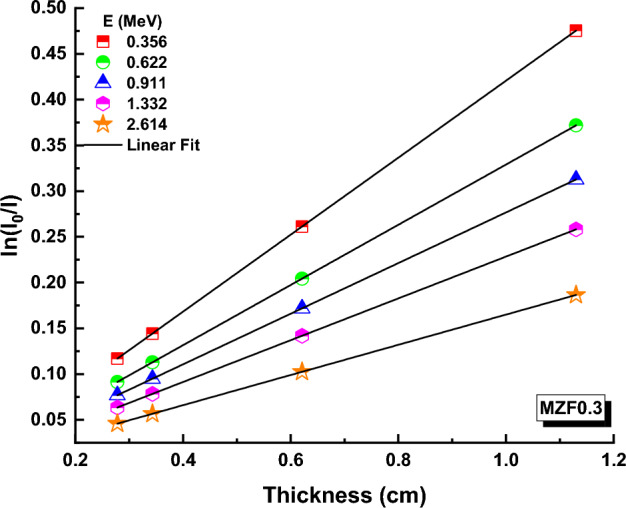
Variation of transmission against thickness-(x) values for MZF0.3 nano-ferrite sample.

**Figure 11:**
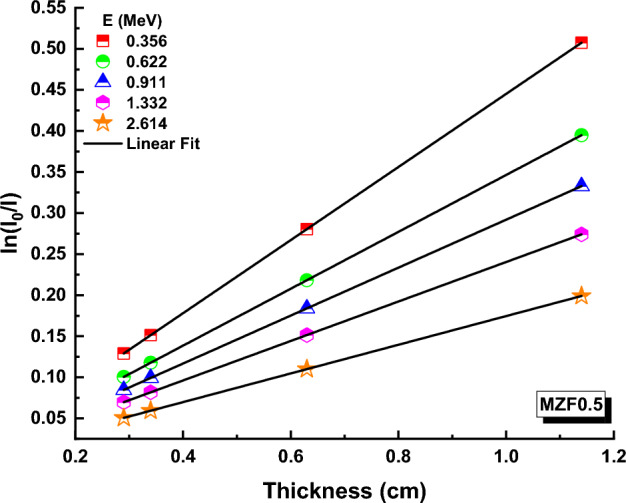
Variation of transmission against thickness (x) values for MZF0.5 nano-ferrite sample.

T values for ferrite samples containing 0.0, 0.10, 0.20, 0.30, 0.40 and 0.50 Zn are depicted in Figs. 9, 10, and 11 at 0.356, 0.662, 0.911, 1.332, and 2.614 MeV. (An example).

T values of ferrite samples fall at a particular energy as Zn content and ferrite samples thickness rise, as illustrated in these figures. T values for MZF0.0, MZF0.3, and MZF0.5 ferrite samples are 0.10012086, 0.116960243, and − 0.12909263 at 0.356 MeV and 0.29 cm, respectively. The Beer–Lambert law can be used to determine the linear attenuation coefficient (µ), which is an important feature for measuring the interaction of photons with ferrite samples^59^:

11 $$\mu =\text{ln}\left(\frac{{I}_{0}}{I}\right)\frac{1}{t}$$

**Figure 12:**
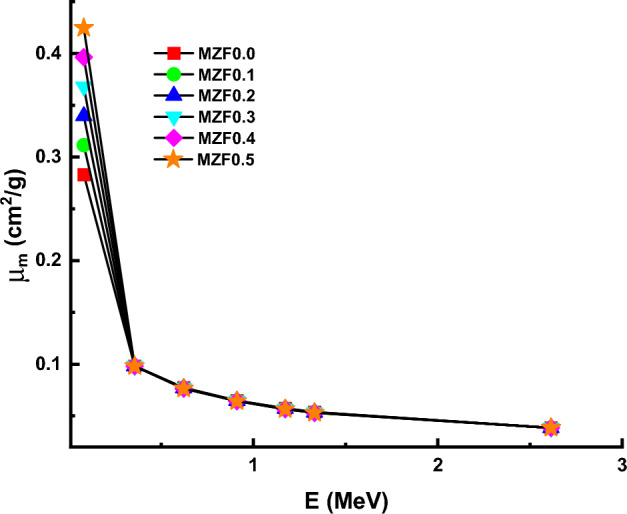
Variation of mass attenuation coefficient (µ_m_) against photon energy for all investigated nano-ferrite samples.

The mass attenuation coefficient (µ_m_) values of for MZF0.0, MZF0.1, MZF0.2, MZF0.3, MZF0.4, MZF0.5 ferrite samples at 0.081, 0.356, 0.662, 0.911, 1.173, 1.332, and 2.614 MeV are shown in Fig. 12. With rising photon energy, the values fall. Photons interact with matter in three different ways, depending on their energy. When it comes to interactions, the photoelectric effect, Compton scattering, and pair creation are all phenomena that occur at different energy levels: low, medium, and high, respectively.

**Figure 13:**
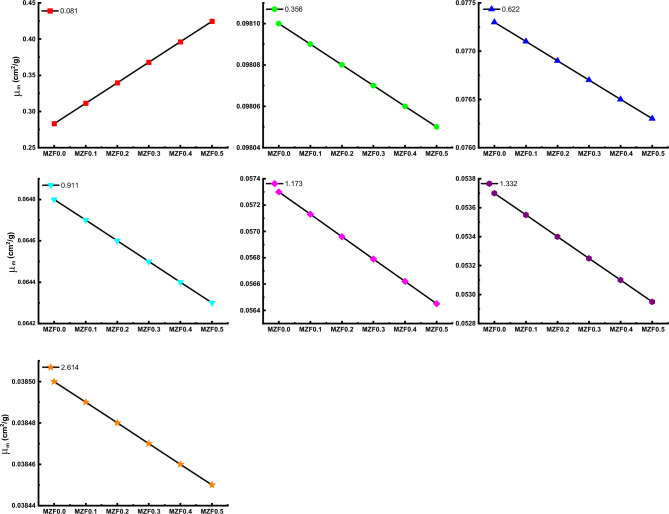
Variation of mass attenuation coefficient (µ_m_) against investigated nano-ferrite samples.

At 0.081, 0.356, 0.662, 0.911, 1.173, 1.332, and 2.614 MeV, values versus ferrite composition are shown in Fig. 13. There was an exception to this rule in Fig. 13, where mass attenuation values for all samples except for that at 0.081 MeV decrease as Zn content increases from 0 to 0.5 wt%. This may attribute to dominate the Compton scattering in this energy region. Where the probability of a Compton reaction occurring is proportional to Z and photon energy (E) according to Z/E.

Radiation shielding design relies heavily on the (T_0.5_) half-value layer. The thickness of the material required to reduce the incident photon intensity to 50% of its starting value is referred to as this characteristic^60^:

12 $${T}_{0.5}=\frac{\text{ln}(2)}{\mu }$$

**Figure 14:**
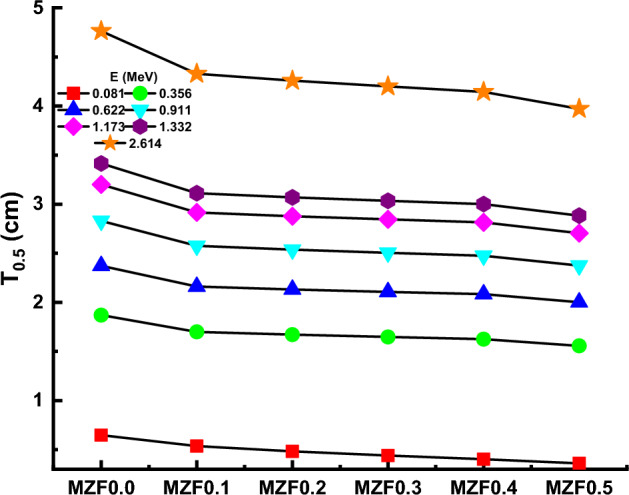
Variation of half-value layer (T_0.5_) against photon energy for all samples.

The T_0.5_ values of the ferrite samples at 0.081, 0.356, 0.662, 0.911, 1.173, 1.332, and 2.614 MeV have been measured and plotted in Fig. 14. Ferrite samples were found to have lower T_0.5_ values when Zn content increased from 0 to 0.5 wt%. For example, at 0.356 MeV, 1.87, 1.70, 1.67, 1.65, 1.62, and 1.56 cm are the T_0.5_ values of the MZF0.0, MZF0.1, MZF0.2, MZF0.3, MZF0.4, and MZF0.5 ferrite samples, respectively. Also, the T_0.5_ values of all ferrite samples increase as the photon energy increase. For MZF0.5 sample, 0.36, 1.56, 2.00, 2.37, 2.70, 2.88, and 3.97 cm are the T_0.5_ measured values at 0.081, 0.356, 0.662, 0.911, 1.173, 1.332, and 2.614 MeV. The results show that the MZF0.0 and MZF0.5 ferrite samples have the highest and lowest T0.5 values, respectively.

## Conclusion

Magnesium Zinc ferrite was successfully synthesized using the Co-precipitation method and characterized using XRD and FTIR techniques. The XRD patterns confirm the formation of a single phase. XRD data was employed to explore structural properties such as Lattice parameter a_exp_ (Å), crystallite size t (nm), interplanar distance d (nm), X-ray density d_x_ (g/cm^3^), Bulk density d_B_ (g/cm^3^), Porosity P (%), Interchain separation R (nm), microstrain (ɛ), dislocation density δ (nm^-2^), and distortion parameters (g). it was found strongly depending on structural parameters with replacement Zn with Mg ions. From FTIR spectra, both ν_1_ and ν_2_ vibration frequencies for tetrahedral and octahedral sites increased in the range of 609–624 cm^−1^ and 461–482 cm^−1^, respectively, which further employed to calculate force constants. The magnetic hyperfine field and isomer shift strongly depending on Zn in the Mg-ferrite composition. Adding Zn to Magnesium Zinc ferrite MZF-nano-ferrite enhanced density and improved the gamma shielding properties. The µm properties were determined experimentally at 0.081, 0.356, 0.662, 0.911, 1.332, and 2.614 MeV. The gamma shielding properties for the MZF-nano-ferrite sample are highest compared with other samples at low energy. For example, the MAC values at 0.081 MeV are 0.283, 0.311, 0.340, 0.368, 0.396, and 0.425 cm^2^/g for MZF0.0, MZF0.1, MZF0.2, MZF0.3, MZF0.4, and MZF0.5 ferrite samples; while, the MAC values at 2.614 MeV are 0.0385, 0.03894, 0.03848, 0.03847, 0.03846, and 0.03845 cm^2^/g for MZF0.0, MZF0.1, MZF0.2, MZF0.3, MZF0.4, and MZF0.5 ferrite samples. The MZF0.5 results showed superior results for MZF-nano-ferrite at the low-energy and MZF0.0 high-energy. From the obtained results, we can nominate the MZF-nano-ferrite to be a radiation shielding material for γ-rays.

## Data Availability

All data generated or analysed during this study are included in this published article.
