# Author Correction: Structure, Mössbauer, electrical, and γ-ray attenuation-properties of magnesium zinc ferrite synthesized co-precipitation method

**DOI:** 10.1038/s41598-022-26362-0

**Published:** 2022-12-22

**Authors:** Hesham M. H. Zakaly, Shams A. M. Issa, H. A. Saudi, Gharam A. Alharshan, M. A. M. Uosif, A. M. A. Henaish

**Affiliations:** 1 Institute of Physics and Technology, Ural Federal University, Ekaterinburg, Russia 620003; 2 Physics Department, Faculty of Science, Al-Azhar University, Assiut Branch, Assiut, 71524 Egypt; 3 Physics Department, Faculty of Science, University of Tabuk, Tabuk, 71451 Saudi Arabia; 4 Department of Physics, Faculty of Science, Al-Azhar University (Girls’ Branch), Nasr City, Egypt; 5 Physics Department, College of Science, Princess Nourah Bint, Abdulrahman University, P. O. Box. 84428, Riyadh, 11671 Saudi Arabia; 6 Physics Department, College of Science, Jouf University, P. O. 2014, Sakaka, Al‑Jouf Saudi Arabia; 7 Physics Department, Faculty of Science, Tanta University, Tanta, 31527 Egypt

Correction to: *Scientific Reports* https://doi.org/10.1038/s41598-022-17311-y, published online 15 September 2022

In the original version of this Article M. A. M. Uosif was incorrectly affiliated with ‘Physics Department, Faculty of Science, Al-Azhar University, Assiut Branch, Assiut 71524, Egypt’. The correct affiliation is listed below.

Physics Department, College of Science, Jouf University, P. O. 2014, Sakaka, Al‑Jouf, Saudi Arabia.

The original Article has been corrected.

